# Nipbl Haploinsufficiency Leads to Delayed Outflow Tract Septation and Aortic Valve Thickening

**DOI:** 10.3390/ijms242115564

**Published:** 2023-10-25

**Authors:** Fanny Boulet, Gaelle Odelin, Alenca Harrington, Thomas Moore-Morris

**Affiliations:** 1 Institut de Génomique Fonctionnelle, University of Montpellier, Centre National de la Recherche Scientifique, Institut National de la Santé et de la Recherche Médicale, 34094 Montpellier, France; 2 Blizard Institute, Barts and The London School of Medicine and Dentistry, Queen Mary University of London, London E1 2AT, UK; 3 Aix Marseille University, INSERM, MMG, 13005 Marseille, France

**Keywords:** Nipbl, cardiac valves, neural crest

## Abstract

Cornelia de Lange Syndrome (CdLS) patients, who frequently carry a mutation in NIPBL, present an increased incidence of outflow tract (OFT)-related congenital heart defects (CHDs). *Nipbl+/-* mice recapitulate a number of phenotypic traits of CdLS patients, including a small body size and cardiac defects, but no study has specifically focused on the valves. Here, we show that adult *Nipbl+/-* mice present aortic valve thickening, a condition that has been associated with stenosis. During development, we observed that OFT septation and neural crest cell condensation was delayed in *Nipbl+/-* embryos. However, we did not observe defects in the deployment of the main lineages contributing to the semilunar valves. Indeed, endocardial endothelial-to-mesenchymal transition (EndMT), analysed via outflow tract explants, and neural crest migration, analysed via genetic lineage tracing, did not significantly differ in *Nipbl+/-* mice and their wild-type littermates. Our study provides the first direct evidence for valve formation defects in *Nipbl+/-* mice and points to specific developmental defects as an origin for valve disease in patients.

## 1. Introduction

Congenital heart defects are a predominant cause of mortality and morbidity. In many cases, CHDs involve abnormal cardiac septal and valve tissue formation. Acquired valve diseases increase with age and affect up to 15% of people above 75 years of age [1]. Surgical intervention is frequently required in valvular disease contexts (e.g., aortic stenosis) [2]. Valve replacement presents limitations, notably regarding the durability of bioprosthetic valves [3]. Furthermore, such approaches are problematic in children, for whom valve repair is a preferred strategy [4]. Hence, despite advances in the clinic, identifying the signalling and mechanisms driving valvulogenesis is essential for developing novel therapeutic approaches for preventing and reversing pathological valve remodelling and associated defects.

Valve development depends on the tightly coordinated mobilisation of multiple progenitor populations, including endocardial and neural crest lineages, and the activation of processes such as the endothelial-to-mesenchymal transition (EndMT) [5,6,7]. Such complex morphological events are particularly dependent on epigenetic reprogramming, involving changes to DNA methylation, histone modifications, and genome architecture that enable the fine spatial and temporal control of gene expression. The latter involves the mobilisation of the so-called architectural proteins, including CTCF and the cohesin complex [8,9]. Mutations in such genes can lead to transcription being impacted globally and notably affect early developmental processes dependant on dynamic changes in gene expression [10,11,12].

CdLS is congenital multisystemic genetic disorder associated with pathogenic variants in the cohesin core units (SMC1A, SMC3, RAD21) as well as HDAC8, BRD4, and ANKRD11 [13]; however, up to 70% of cases have been linked to mutations in cohesin loader NIPBL [13,14,15]. Patients present distinctive craniofacial appearance, limb malformations, and cardiac defects and growth. Cardiac defects are observed in 10–70% of patients and do not appear to be specifically linked to mutations in any of the genes mentioned above [13,16,17]. Interestingly, the most common cardiac defects in CdLS patients, including pulmonary stenosis, are valve-associated. Hence, better understanding how NIPBL deficiency affects cardiac morphogenesis, and notably valvulogensis, is an important clinical issue. Little is known about the impact of global cohesin deficiency on the contribution of the various cardiac lineage to outflow tract development and valve formation. Previous studies in mice have reported up to 30% atrial–septal defects in *Nipbl+/-* mice [10,18]. In zebrafish, the knockdown of the cohesin complex member Rad21 prevented neural crest migration to the heart, suggesting that this lineage could play a key role in the defects observed in patients [19]. However, the conditional deletion of *Nipbl* in the neural crest in mice did not cause a cardiac phenotype [20].

In the current study, we found delayed OFT septation in *Nipbl+/-* mice that was linked to the delayed condensation (but not migration) of neural crest cells. In adulthood, despite normal functional parameters, *Nipbl+/-* mice presented aortic valve thickening. These findings provide the first direct evidence for valve development defects in *Nipbl+/-* mice and suggest that valve-related cardiac defects in CdLS patients could result from delayed OFT septation and valve thickening. 

## 2. Results

### 2.1. Aortic Valve Thickening in Nipbl+/- Mice

**Figure 1:**
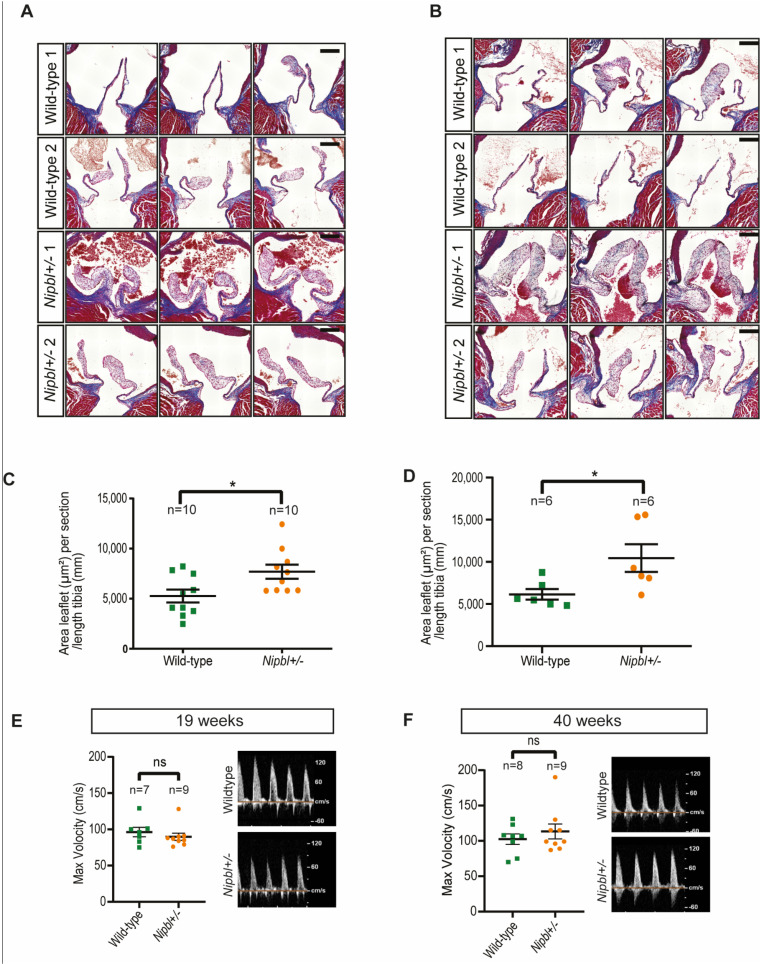
Thickening of aortic valves in *Nipbl+/-* mice. (**A**,**B**) Trichrome-stained consecutive sections of aortic valves from two representative adult male wild-type (WT) and *Nipbl+/-* mice at 19 weeks (**A**) and 40 weeks (**B**). Scale bars: 200 µm. (**C**,**D**) Quantification of average aortic valve area over tibia length at 19 weeks (**C**) and 40 weeks (**D**). (**E**,**F**) Echographic analysis of aortic valve function in adult wild-type control and *Nipbl+/-* mice at 19 weeks (**E**) and 40 weeks (**F**). Insets show representative Doppler echocardiographic images. Mean + SEM (* *p* < 0.05, ns *p* > 0.05, two-sided and unpaired *t* tests).

We confirmed that the Nipbl transcript was reduced approximately two-fold in *Nipbl+/-* embryos and that adult mice presented a significant reduction in bodyweight, as previously reported [20] (Supplemental Figure S1). In order to evaluate aortic and pulmonary valve morphology in the *Nipbl+/-* mice, we performed histological analyses. As reported previously for another *Nipbl+/-* line [18], gross anatomical appearance was normal. However, a detailed histological analysis of the aortic and pulmonary valves revealed a thickening of the aortic valve leaflets in adult mice at 19 and 41 weeks of age (Figure 1A,B). Quantification revealed that aortic valve thickening (AVT) was significant in mutant mice (Figure 1C,D). Conversely, we did not observe any thickening of the pulmonary valve leaflets (Supplemental Figure S2). 

In order to assess whether this remodelling of the leaflets affected valve function, we performed Doppler echocardiographic analysis on 19-week and 40-week old mice. We did not detect a significant difference in the maximum velocity of aortic or pulmonary flux (Figure 1E,F; Supplemental Figure S2) or valve insufficiency. Notably, one *Nipbl+/-* mouse presented aortic stenosis at 40 weeks (Figure 1F), and one wild-type mouse presented pulmonary stenosis (Supplemental Figure S2).

Hence, although the *Nipbl+/-* mice presented AVT, this was not associated with a significant difference in the maximum velocity of aortic flux. AVT without aortic stenosis (AS) is common in ageing patients and has been associated with the development of AS [21]. 

### 2.2. Normal Endothelial-to-Mesenchymal Transition in Nipbl+/- Outflow Tracts

**Figure 2:**
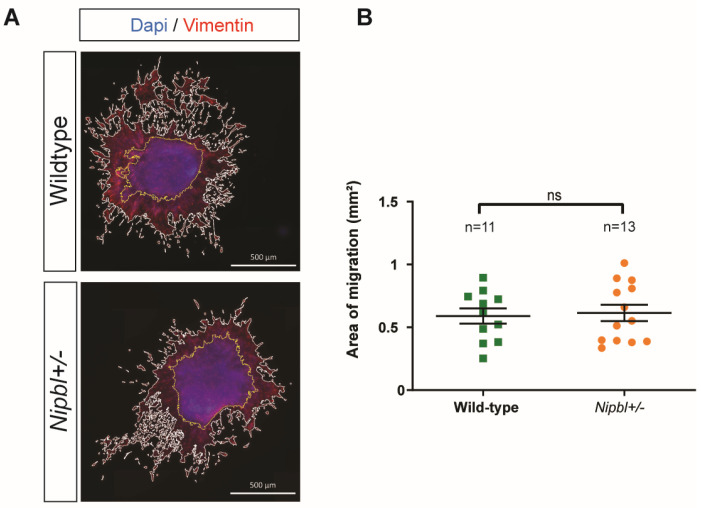
Normal EndMT in *Nipbl+/-* OFT explants. (**A**) Immunofluorescence staining of outflow tract (OFT) explants. Vimentin, red; DAPI, blue. The area of migration (white line) and explant area (yellow line) were defined using imaging software (ImageJ.org). Scale bars: 500 µm. (**B**) Comparison of the area of migration from explants from wild-type and *Nipbl+/-* OFTs. Mean + SEM (ns *p* > 0.05, two-sided and unpaired *t* tests).

In order to investigate whether early developmental defects could underlie the pathological remodelling observed in the aortic valve, we focused on the early processes leading to valvulogenesis, including endocardial endothelial-to-mesenchymal transition (EndMT) [22]. For this, we used an E10.5 OFT explant assay to compare EndMT and the migration of endocardial valve progenitors from *Nipbl+/-* embryos to that of their wild-type counterparts (Figure 2A). Explants from both genotypes displayed clear EndMT, and measuring mesenchymal cell migration did not reveal any significant difference between the mutant and wild-type explants (Figure 2B). These results suggested that the OFT valve progenitor’s propensity to undergo EndMT and migrate was not affected by Nipbl haploinsufficiency. 

### 2.3. Cardiac Neural Crest Cell Condensation Is Delayed in Nipbl+/- OFTs

**Figure 3:**
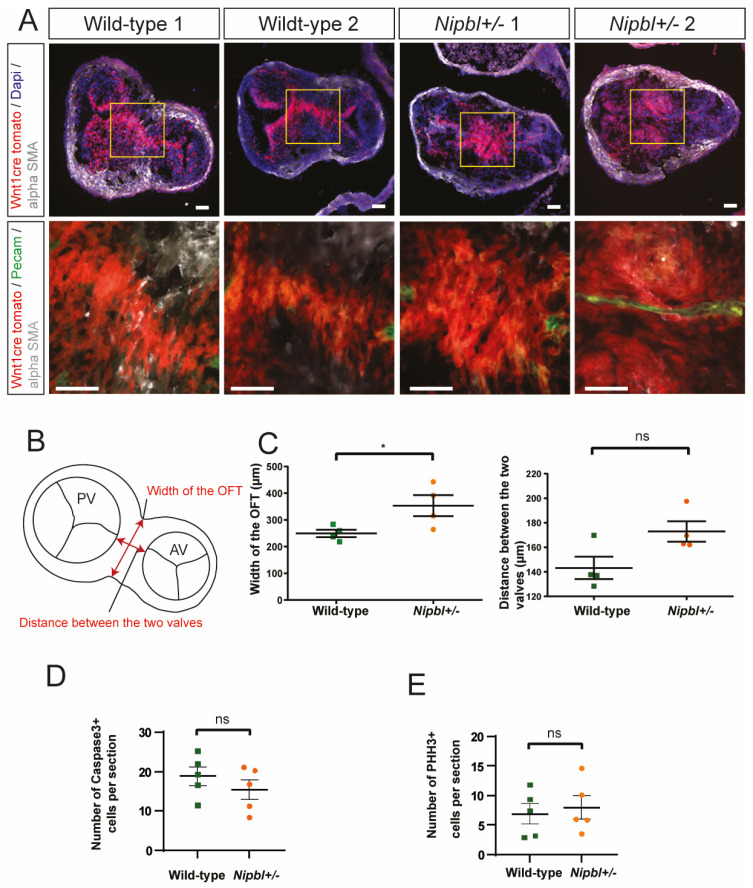
Delayed OFT septation and neural crest cell condensation in *Nipbl+/-* OFTs. (**A**) Representative immunofluorescence-labelled mid-sections of *Wnt1-Cre+/-*; *ROSA-tdT+/-* and *Nipbl+/-*; *Wnt1-Cre+/-*; *ROSA-tdT+/- E12.5* OFTs. The tdT+ neural crest cells are visibly less condensed in the *Nipbl+/-* background. Scale bars: 50 µm. (**B**) Illustration indicating the parameters measured in the OFTs. (**C**) Quantification of OFT width and distance between the aortic and pulmonary valves in the *Nipbl+/-* and wild-type E12.5 OFTs. (**D**) Quantification of caspase-3+ cells in OFTs at E12.5. (**E**) Quantification of proliferating (Phosphohistone H3+ (pHH3+) cells in OFTs at E12.5. Mean + SEM (* *p* < 0.05, ns *p* > 0.05, two-sided and unpaired *t* tests).

The cardiac neural crest specifically contributes large numbers of mesenchymal cells to the OFT and plays a key role in OFT septation, which is required for the formation of the aortic and pulmonary valves [5,7]. As cardiac defects in cohesin-deficient zebrafish have been linked to a lack of neural crest migration [19], we performed genetic lineage tracing for the neural crest cells in the *Nipbl+/-* mice. To achieve this, we generated and analysed *Wnt1-Cre+/-;ROSA-tdT+/-*;*Nipbl+/-* embryos at different developmental stages. In contrast to the observations made in the zebrafish study [19], we found that neural crest migration occurred normally and that neural crest cells were abundant in the outflow tracts of *Wnt1-Cre+/-;ROSA-tdT+/-;Nipbl+/-* embryos (Figure 3, Supplemental Figure S3). However, we did observe a delay in neural crest cell condensation (Figure 3A), which was associated with an increase in mid-sectional OFT width (Figure 3B,C). The remodeling associated with septation has been reported to involve active proliferation and apoptosis [23]. However, we did not detect significant differences in the proliferation or apoptosis of *Wnt1-Cre+/-;Rosa-tdT+/-* neural crest cells between the *Nipbl+/-* and wild-type embryos in OFT (Figure 3D,E).

## 3. Discussion

In this study, we provide the first evidence for valve defects in a *Nipbl+/-* murine model of CdLS. We observed a thickening of the aortic valve cusps in young and old adult mice that, at the stages investigated, was not significantly associated with stenosis or abnormal valve function. Strikingly, although neural crest migration did not appear to be affected, OFT septation and neural crest condensation were delayed in the *Nipbl+/-* embryos.

CdLS is congenital multisystemic genetic disorder with a prevalence of approximately 1/10,000–1/30,000 live births [13,17]. At least half of the cases are linked to *NIPBL* mutations [13,16]. OFT-related CHDs, including pulmonary and aortic stenosis, are among the most common CHDs observed in CdLS patients [16]. We observed a thickening of the aortic valve leaflets, but not pulmonary valve leaflets, in adult *Nipbl+/-* mice. In addition, we detected aortic stenosis (AS) in one of eight 41-week-old adult mice but none in wild-type controls. AVT has been identified as a significant risk factor for aortic disease, including AS [21]. Hence, our study indicates that *Nipbl+/-* mice may be predisposed to AS resulting from AVT. Semilunar valve thickening has been associated with stenosis in CdLS patients, but in human patients, it is more frequently observed for the pulmonary valve [24]. Notably, another study indicated that approximately 4–5% of CdLS patients presented pulmonary stenosis [16]. Aortic stenosis was less frequent, but aortic valve defects, including dysplasia, were common among cardiac defects [16].

CdLS, typically associated with a germline mutation in *NIPBL*, is a developmental syndrome [16,25]. Hence, we investigated the properties of the key lineages involved in semilunar valve development, including the endocardium and the neural crest. OFT explant culture represents a powerful ex vivo approach for evaluating endocardial EndMT [26,27]. Following culture, *Nipbl+/-* OFT explants were indistinguishable from those of the wild-type controls, suggesting that EndMT and the migration of the mesenchymal cells produced was not affected in mutant mice. Although the neural crest directly contributes to OFT development, several studies have reported that the conditional deletion of *Nipbl* in neural crest does not produce a phenotype in mice [18,20]. This implies that the *Nipbl* deficiency within the neural crest lineage was insufficient to cause CHDs but does not address the issue of how global haploinsufficiency could impact neural crest deployment and neural crest cells’ contribution to valve formation. Our data show that *Nipbl* haploinsufficiency led to delayed neural crest compaction in the aortopulmonary septum (associated with delayed OFT septation). We did not observe any effect on neural crest cell migration into the OFT. Furthermore, we could not detect differences in cell proliferation or apoptosis in E12.5 OFTs, although we cannot rule out differences at earlier stages or, considering variability, with larger sample sizes. A previous study reported that cohesin deficiency in zebrafish leads to impaired neural crest migration and valve defects (summarised in Supplemental Table S1) [19]. The cohesin complex and its partners are highly conserved among species. Notably, zebrafish and mouse Nipbl proteins share a very similar structure, including multiple HEAT domains (Supplemental Figure S4). Beyond potential inter-species differences, these conflicting results may be due to the fact that this previous study in zebrafish targeted cohesin complex component *rad21*, suggesting that cohesin deficiency and Nipbl deficiency do not necessarily go hand in hand. This would be in line with a previous study predicting a “cohesin-independent role” for Nipbl [28]. However, a more likely cause for the discrepancies between our studies is that Schuster et al. [19] relied on morpholinos that produced a transient decrease in Nipbl levels. Also, knockdown, as opposed to genetic knockout, does not allow for potential RNA decay-mediated compensation [29] that could result in a less pronounced lack of migration. Such compensatory mechanisms could be at play in the *Nipbl+/-* mouse model employed in this study, with one allele lacking start codon-containing exon 2 but expressing a truncated Nipbl transcript [20].

As Nipbl deficiency leads to global transcriptional misregulation, it will be challenging to pinpoint defects in the specific genes and pathways that underlie the phenotypes observed in adult *Nipbl+/-* mice and more generally in CdLS patients and models. Several genes have been linked to semilunar valve thickening. Notably, mutations in GATA4, expressed in multiple mesenchymal and endothelial valve cell populations, have been associated with semilunar valve thickening in patients, and this was faithfully reproduced in GATA4 mutant mice [30]. In the neural crest, Pax3 deficiency has been shown to cause semilunar valve thickening [31]. Several genes and signalling pathways have been linked to OFT septation. Notably, conditional deletion experiments in mice have shown that signalling between the neural crest and endothelium via Sema3C secretion and NRP1 expression, respectively, is essential for OFT septation [32]. The mesodermal expression of BMP4 is also key to ensure proper septation [33]. Hence, normal OFT septation requires specific signalling in several lineages, which would be affected by the global perturbations resulting from Nipbl haploinsufficiency. 

Although cardiac defects in CdLS patients are variable and not systematic, there is a clear tendency towards the pulmonary (rather than aortic) stenosis [16]. Our study results reveal AVT in adult *Nipbl+/-* mice, rather than pulmonary-valve associated defects. This could be linked to differences in gene dosage between mice and humans with Nipbl deficiency. On the other hand, AVT without aortic stenosis in *Nipbl+/-* mice could be representative of isolated valve defects that are frequent in the aortic valves of CdLS patients, including dysplasia [16]. It would be of interest to investigate whether *Nipbl+/-* mice more readily develop aortic valve disease in response to stresses such as pressure overload.

Overall, our data show delayed OFT septation, associated with a lack of neural crest condensation, and AVT in *Nipbl+/-* mice. Early perturbations to OFT development may underlie the AVT observed in adult *Nipbl+/-* mice and the frequent semilunar valve associated CHDs found in CdLS patients. These findings suggest that CdLS patient diagnosis and management should include functional assessments of the semilunar valves.

## 4. Materials and Methods

Mouse lines. *Nipbl+/-* [20], Wnt1-Cre [34], and Rosa-tdT [35] mouse lines were maintained on a CD1 background. Food and water were provided ad libitum.

Echocardiography The mice were anaesthetised using 4% isoflurane and moved to a warming station that maintained core body temperature. The mice were then placed under anaesthesia (1% isoflurane). During the procedure, heart rate, temperature, and respiration were continuously monitored. Ultrasound gel was applied to the chest of the animals, and echocardiography measurements were taken using the Affinity 50 software package and an ultrasound system with a linear transducer (Philips, Amsterdam, Netherlands). Doppler imaging was obtained from 19-week-old and 40-week-old mice. A flow >2 m·s^−1^ was considered to be indicative of stenosis.

OFT explants. OFTs were dissected from E10.5 embryonic hearts, cut open, and cultured cushion-side down on collagen gels as previously described [36]. Briefly, OFTs were opened and placed endocardial-side down on collagen type I gels (collagen type I from rat tails—EMD Millipore (Burlington, MA, USA), 10× DMEM—Invitrogen (Waltham, Massachusetts, United States), NaOH 1M, H20) overnight in a cell culture incubator. The following day, culture media (DMEM 10% foetal calf serum, Insulin-Transferrin-Selenium—Sigma (St. Louis, Missouri, United States), Penicillin-streptomycin—(Invitrogen) were added. Following culture, the explants were then fixed overnight in 4% PFA, permeabilised overnight in PBS with 0.2% triton, and subjected to immunostaining. Primary and secondary antibody labelling steps were carried out overnight at 4 degrees. Image stacks were generated on an Apotome^®^ fluorescence microscope, and quantifications were performed in Zen Blue edition (Zeiss, Oberkochen, Baden-Württemberg, Germany).

Tissue preparation. Adult mouse hearts were dissected, washed in PBS, fixed in 4% PFA overnight, paraffin-embedded, and sectioned (5–10 µm). Briefly, embryos were dissected, fixed in 4% PFA overnight, dehydrated in 20% sucrose, embedded in OCT/sucrose, and sectioned (10 µm). 

Tissue staining. Trichrome staining was performed using a Trichrome Stain (Masson) kit (HT15, Sigma) following the specifications and instructions of the manufacturer. For haematoxylin and eosin staining, slides were incubated for 2 min in a haematoxylin solution, followed by 30 s differentiation before being washed in water. The slides were then counterstained for 2 min in eosin, dehydrated in ethanol, rinsed in xylene, and mounted.

Immunofluorescence staining. Immunostaining was performed as previously described [37]. Briefly, the sections were washed in PBS, permeabilised in PBS + 0.1% triton, and then incubated for 1 h in blocking solution (PBS, 10% foetal bovine serum). The slides were then incubated with the primary antibody in blocking solution overnight, washed, and incubated with the secondary antibody and DAPI in blocking solution for 90 min before being washed in PBS and mounted using Fluoromount. 

qPCR. RNA was extracted from whole E9.5 embryos using a Quick RNA mini prep kit (Zymo Research, Beijing, China), and RT was performed using Superscript III Reverse transcriptase (Invitrogen) according to the manufacturer’s instructions. qPCR was performed using LightCycler 480 SYBR Green I Master and the LightCycler 480 system (Roche, Basel, Switzerland). mRNA levels were normalised against HRPT as a housekeeping gene, and fold changes were calculated using the log 2ΔΔCt method.

Primers:
Nipbl:
Fwd: GCCGATTCGCCCAGAGTTT
Rev: CCTGAAGTTCTGGAATGGTGT
HPRT:
Fwd CTG GTG AAA AGG ACC TCT CG
Rev TGG CAA CAT CAA CAG GAC TC

Antibodies

**Antigen**

**Antibody**

**Dilution**
VimentinAbcam, ab1398781/100PecamBD pharmigen, 5502741/100αSMAAbcam, ab56941/200Phospho-Histone H3CST, 3377T1/500Cleaved Caspase-3CST, 9661T1/500

Protein sequence alignment. Protein sequences were obtained from NCBI and aligned using T-Coffee in Snapgene (Version 6.1.2).

## Data Availability

Data presentation and the statistical tests used are described in the figure legends.

## Supplementary Materials

The following supporting information can be downloaded at: https://www.mdpi.com/article/10.3390/ijms242115564/s1.
