# Supplementary material for: Nipbl Haploinsufficiency Leads to Delayed Outflow Tract Septation and Aortic Valve Thickening
Source: Int J Mol Sci. 2023 Oct 25;24(21):15564. doi: 10.3390/ijms242115564 (PMC10648932; doi:10.3390/ijms242115564)
Supplement: Supplementary file 1 [file ijms-24-15564-s001.zip › ijms-2614130-Supplementary Materials.pdf]

Supplementary Materials

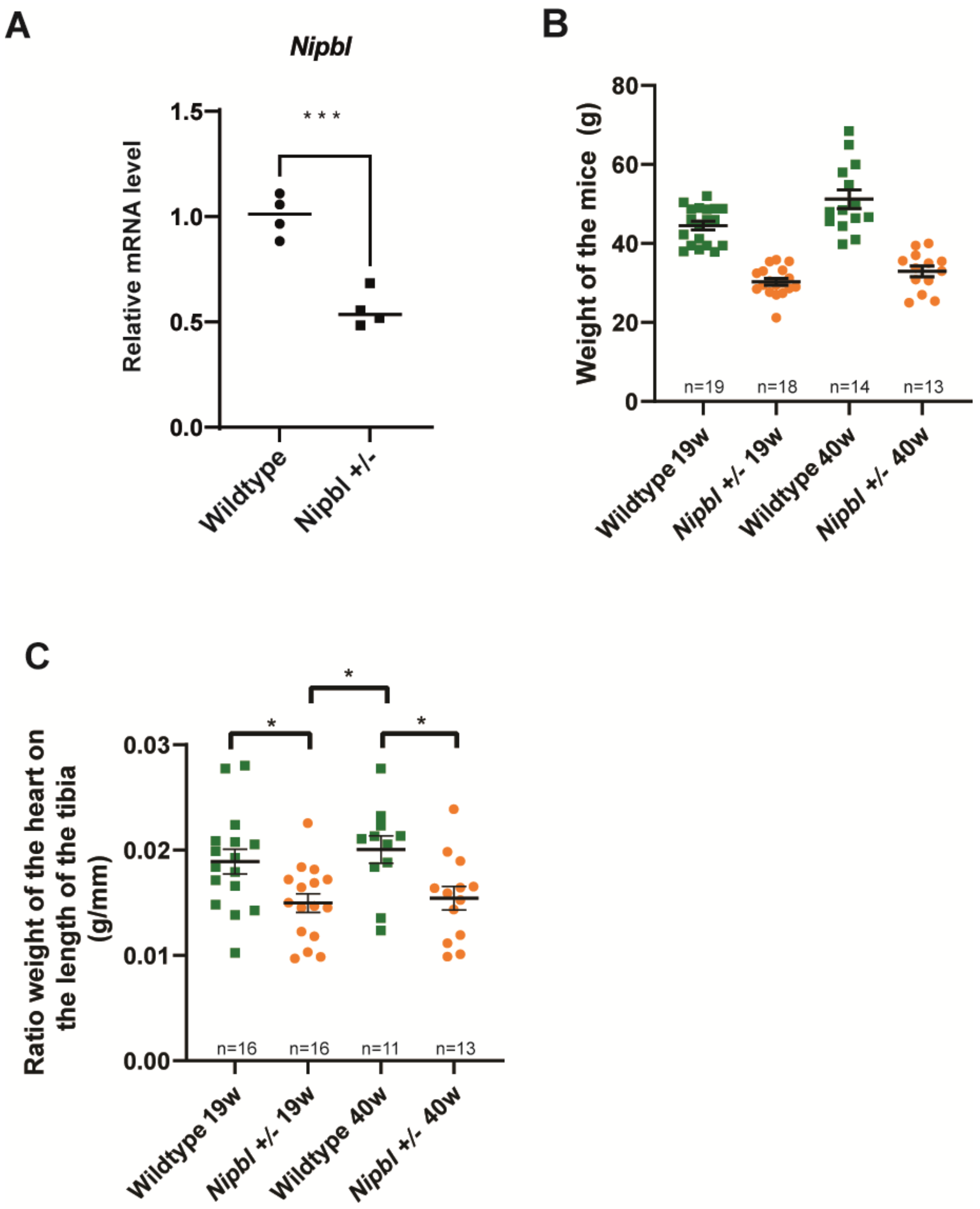

Supplemental Figure S1. A *Nipbl* transcript levels in whole E9.5 embryos. ***p<0.001, two-sided and unpaired t test. **B** Weight in grams of *Nipbl+/-* and wild-type littermate controls at 19 and 40 weeks. **C** Heart weight over tibia length ratios at 19 and 40 weeks. *p<0.05, one way ANOVA with multiple comparisons.

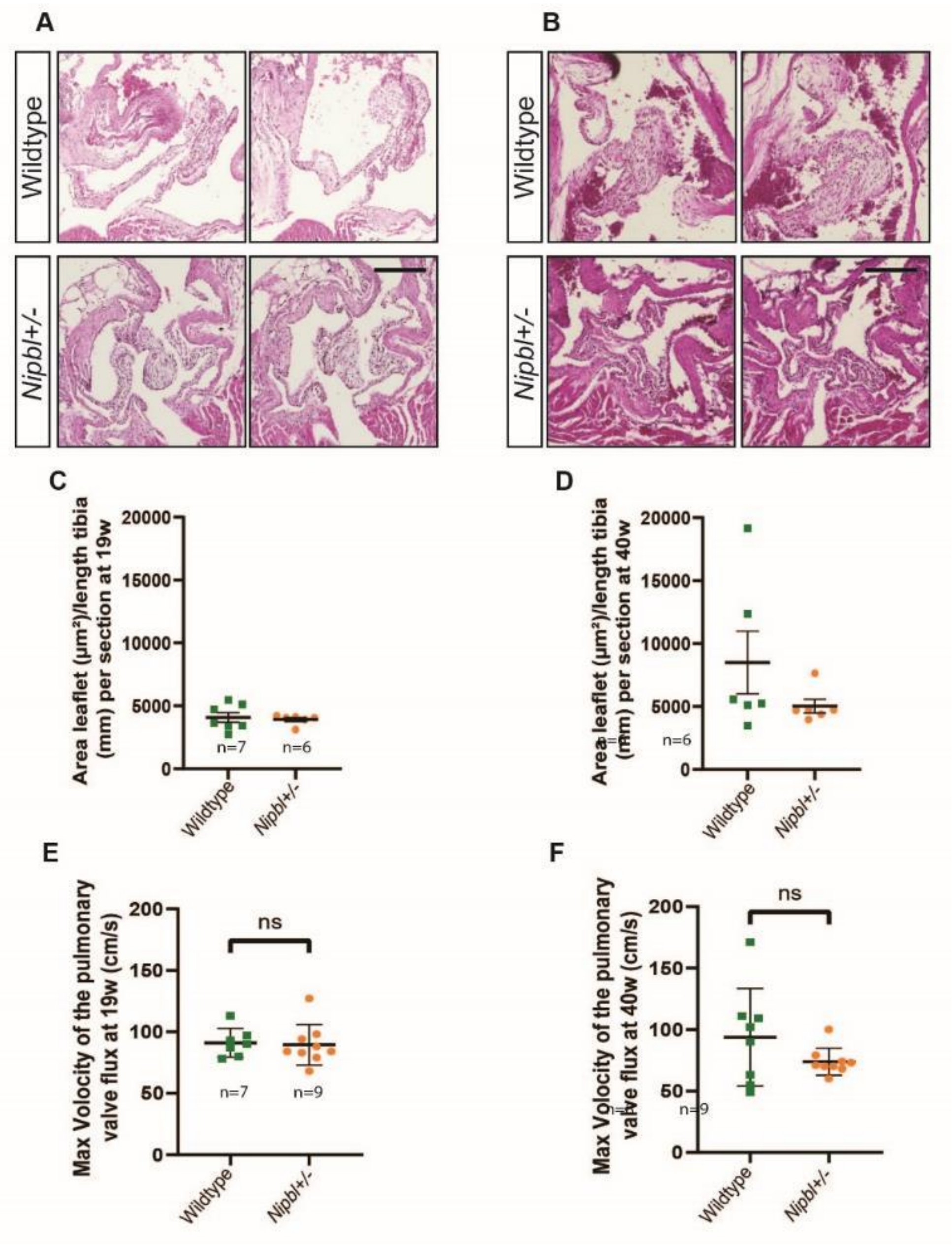

Supplemental Figure S2. A-B. Hematoxylin eosin stained sections of pulmonary valves from representative 19 and 40 week-old male wild-type (WT) and *Nipbl+/-* mice. **C-D.** Quantification of average pulmonary valve area over tibia length at 19 and 40 weeks. **E-F** Echocardiographic assessment of pulmonary valve function in adult wild-type control and *Nipbl+/-* mice at 19 and 40 weeks. ns = not significant, $p > 0.05$; two-sided and unpaired t test. Scale bar 200µm.

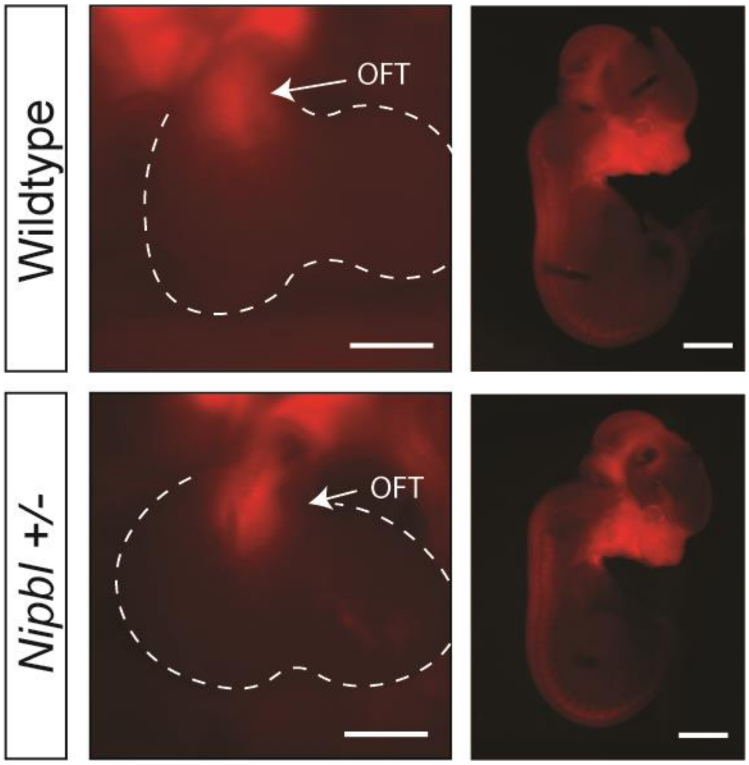

Supplemental Figure S3. Neural crest cell migration in *Wnt1-Cre+/-;ROSA-tdT+/-* and *Nipbl+/-;Wnt1-Cre+/-;ROSA-tdT+/-* embryos. Images show tdT+ (red) neural crest cell distribution in whole-mount heart (center) and whole-mount embryos (right). Dotted lines indicate the contour of the ventricles. Scale bars are 0.25mm (left) and 0.5mm (right).

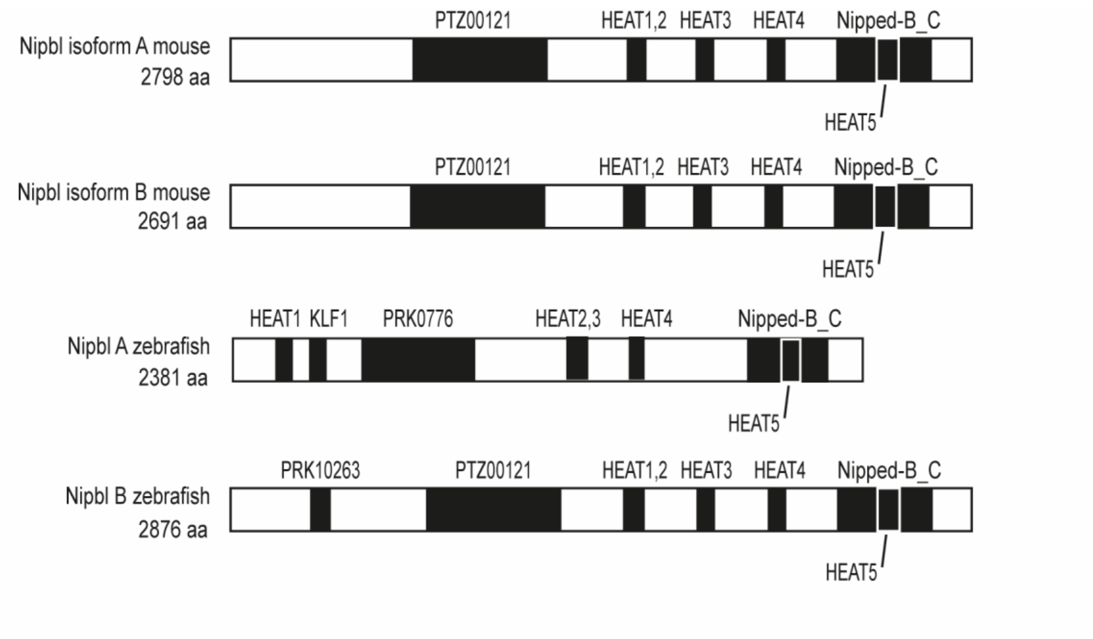

Supplemental Figure S4. Nipbl protein architecture conservation in mice and zebrafish. There is a strong conservation of the C-terminal Nipped-B_C domain. C-terminal HEAT domains are involved in cohesin binding.

	**Current study**	***Schuster et al. [19]***
**Mutant model species**	Mouse	Zebrafish
**Approach for cohesin targeting**	Genetic deletion of Nipbl Exon 2	Morpholino knock down of Rad21
**Neural crest invasion of heart**	Normal	Neural crest cells present but unable to invade the heart
**Defects in valve morphogenesis**	Delayed outflow tract septation	Delay or absence of AV and/or VB development
**Effects on valve progenitor proliferation/apoptosis**	None detected	Not assessed
**Endocardial EMT**	Normal	Not assessed
**Adult valve defects**	Thickening of aortic valve	Not assessed*

AV, atrioventricular valve; VB, ventriculobulbar valve. * due to early lethality.

Supplemental Table S1. Comparison phenotypic changes observed during outflow tract development in *Nipbl+/-* mice and following cohesin knockdown in zebrafish.
